# A simple route to a novel acid-sensitive 20(*S*)-*O*-linked camptothecin norcantharidin acid ester derivative

**DOI:** 10.1098/rsos.170842

**Published:** 2018-02-14

**Authors:** Changkuo Zhao, Xianheng Wang, Fuhong Yang, Lei Gao, Yuhe Wang

**Affiliations:** 1 Department of Pharmacy, Zunyi Medical University, Zunyi, 563003, People's Republic of China; 2 Department of Pharmacy, Zunyi Medical University Affiliated Hospital, Zunyi, 563003, People's Republic of China

**Keywords:** camptothecin, norcantharidin, synthesis, water solubility, tumour

## Abstract

A facile synthetic method was developed for a novel acid-sensitive camptothecin norcantharidin acid ester derivative **I**. The total yield can reach 71%. This method provides several advantages, including high yield and simple working procedure for the synthesis of analogues. The new synthetic compound **I** has been shown to exhibit better solubility and similar activity against tumour cell lines.

## 1. Introduction

**Figure 1.**
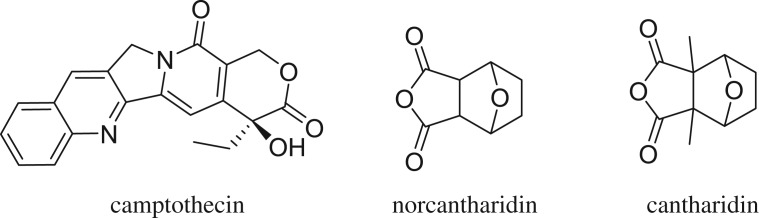
Structure of camptothecin, cantharidin and norcantharidin.

Camptothecin was first isolated by Wall in 1966 from the Chinese tree *Camptotheca acuminate* (figure 1). This isolated compound has a pentacyclic ring system with only one asymmetrical cent in ring E with a 20(*S*)-configuration. This pentacyclic ring system includes a pyrrol [3,4-b] quinoline moiety (rings A, B and C), a conjugated pyridine (ring D) and a six-membered lactone (ring E) with an α-hydroxyl group. However, the initial clinical studies showed that camptothecin was not usable as an anti-cancer agent *in vivo* due to its high toxicity [1–3].

Cantharidin is a naturally occurring toxin which has been isolated in the ‘Spanish fly' *Cantharis vesicatoria*, and then found in many related Mylabris species. Despite potent activity against different cancer cell lines, the nephrotoxicity of cantharidin has prevented it from entering mainstream oncology. Norcantharidin, the demethylated analogue of cantharidin, has a unique feature which observed during clinical trials: the stimulation of the bone marrow production of white cells, which is in contrast to most other anti-cancer drugs that readily induce myelosuppression [4–6].

Many medicinal chemists have been paying much attention to camptothecin due to its noteworthy activity in the mouse leukaemia L 1210 system. A number of CPT derivatives were synthesized and some of them have been used in different preclinical and clinical stages. Among these analogues, 20(*S*)-*O*-linked camptothecin esters offer several advantages, including the improvement of solubility, stability, pharmacokinetics and toxicity [7–10].

**Figure 2.**
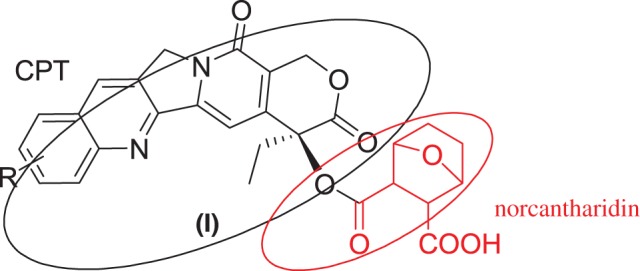
Proposed molecule combined with camptothecin and norcantharidin.

In our present high-throughput screen project for new anti-cancer drugs, a new molecule that combined CPT with norcantharidin in ester-bond way was proposed, which was expected to have good water solubility and better activity against tumour cell lines. Herein, we report the synthesis of this unique molecule in figure 2.

## 2. Results and discussion

Camptothecin and norcantharidin are both commercially available materials and are, therefore, chosen as starting materials for the preparation of the target molecule (**I**). WO 2008021015 provides a general acid-catalysed process for the preparation of 20(*S*)-*O*-linked ester. However, one of our reaction substrates, norcantharidin, is highly acid sensitive and subject to ring-opening.

**Scheme 1.**
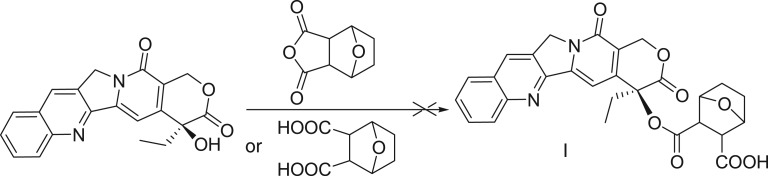
Coupling of CPT with norcantharidin or its acid.

Then we attempted to couple camptothecin and norcantharidin or its acid directly under reflux condition in the presence of EDCI and DMAP and DCMas solvent. Neither of them offers any desired product (scheme 1).

**Scheme 2.**
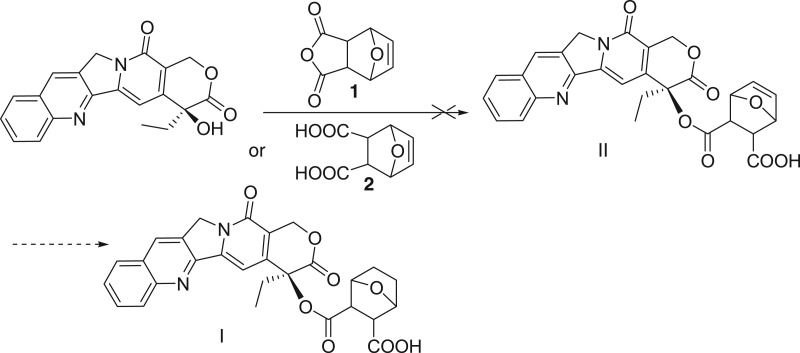
Coupling of CPT with 5-ene-norcantharidin **1** or its acid **2**.

And then another route is proposed to synthesize the target compound (scheme 2). Under the same conditions, camptothecin reacts with some more electrophilic acid, unsaturated 5-ene norcantharidin **1** or its acid **2**, but does not form the desired product at all.

Based on the above results, it is very difficult to directly link CPT to the dicarboxylic acid under normal conditions, which may be due to the high polarity, low solubility, low electrophilicity and space barrier. Therefore, the coupling substrate was replaced by introducing a protecting group in order to reduce its polarity and increase its affinity.

**Scheme 3.**
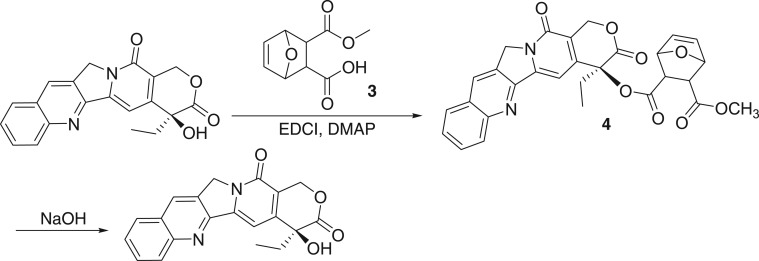
Choosing methyl as a protecting group.

First, 5-ene-norcantharidine monomethyl ester **3** was selected as the coupling substrate. We were surprised that the coupling of CPT to compound **3** was carried out well with the expected yield of compound **4**. However, in the following hydrolysis, the ester **4** is easily cleaved to form CPT and no desired product is obtained (scheme 3).

**Scheme 4.**
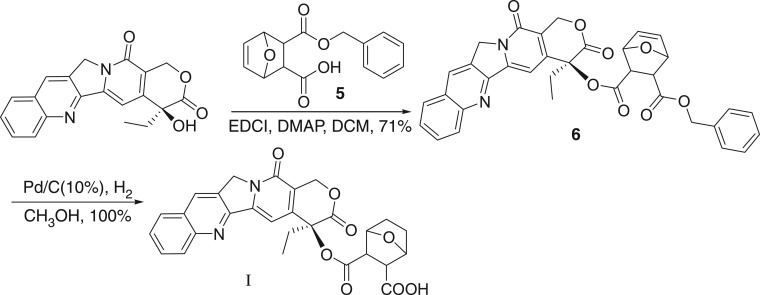
Choosing benzyl as a protecting group.

Then we try to use benzyl as a protecting group. Thus, the acid anhydride **1** is treated with benzyl alcohol to give the desired 5-ene-norcantharidin monobenzyl ester **5** in an excellent yield. In the following, monobenzyl ester **5** was coupled with CPT in a sealed tube at 50°C in the presence of EDCI and DMAP and DCM as a solvent to give the desired product **6** in 71% yield. The resulting benzyl-protected unsaturated ester **6** is then easily hydrogenated under the hydrogen moiety catalysed by Pd/C (10%) to give the target product **I** in a quantitative yield (scheme 4).

## 3. Conclusion

A new acid-sensitive 20(*S*)-*O*-linked CPT norcantharidin acid ester was firstly designed and synthesized from commercially available materials. This protocol gives good reaction yields only when the carboxylic acid is highly electrophilic. At the same time when the acid is less electrophilic, the reaction will give low yield or no expected product at all. This method offers several advantages, including high yield and simple working procedure. It was expected that this unique compound will have a strong activity against different cancer cell lines. The further structure and activity relationship (SAR) study of these derivatives is also underway.

## 4. Experimental section

Melting points were determined by a Mettler Toledo FP 62 melting point apparatus and are uncorrected. ^1^H NMR spectra were recorded at 400 MHz on a Varian Unitynova 400 NMR spectrometer using tetramethylsilane as an internal standard, and then ^13^C NMR spectra were recorded at 100 MHz. Mass spectra were run on a Waters UPLC-MS instrument. UV spectra were determined by a Lambda 25 spectrometer. HPLC spectra were run through a Waters SunfireTM C18, 5 µm, 4.6 mm × 250 mm column on a Waters apparatus (pump: e 2695; detector: 2998). TLC plates (GF 254) were bought from Branch Qingdao Haiyang Chemical Plant. All the solvents and commercial materials are bought from Sinopharm.

### 4.1. Synthesis of side chain 5-ene norcantharidin monomethyl ester **3**


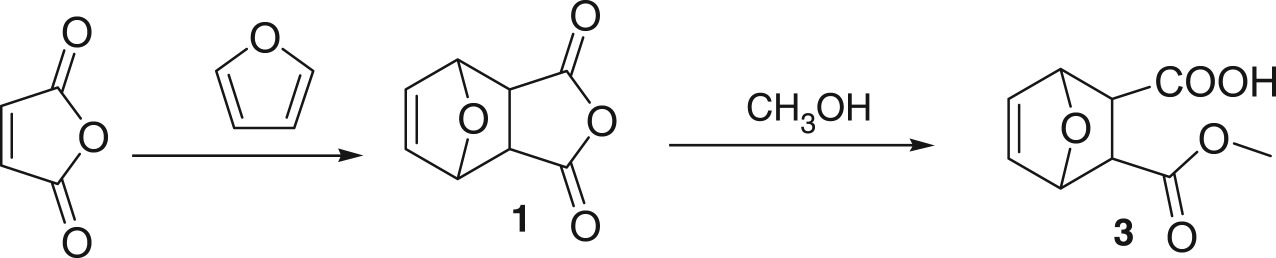


#### 4.1.1. Synthesis of 5-ene-norcantharidin (**1**)

Maleic anhydride (12.02 g) was dissolved in 90 ml of ether. Then 13 ml of furan was added drop-wise in 15 min. The solution was heated to reflux for 1 h and a white crystal was precipitated from the solution. After 24 h, the solid was collected by filtration, dried *in vacuo* to give the product (**1**, 17.46 g, 85.75%). Mp: 122–123°C, *R*_f_: 0.52 (PE: ethyl acetate = 3 : 1); ^1^H NMR (CDCl_3_, 400 MHz): *δ* = 6.58 (s, 2H), 5.47 (s, 2H), 3.18 (s, 2H). ^13^C NMR (DMSO-d_6_, 100 MHz): *δ* = 171.59, 136.88, 81.66, 49.12.

#### 4.1.2. Synthesis of 5-ene norcantharidin monomethyl ester (**3**)

To a suspension of **1** (4.15 g) in 25 ml of methanol was added triethyl amine (0.73 ml). The mixture was stirred at room temperature for 24 h. The solvent was removed under reduced pressure. The crude product was dissolved in 25 ml of dichloromethane and washed with 1 N HCl (7 ml) and brine (10 ml) in sequence. The organic layer was dried over MgSO_4_ to afford the title product (**3**, 4.50 g, 91%) as a white solid. Mp: 110–111°C, ^1^H NMR (DMSO-d_6_, 400 MHz): *δ* = 12.51 (br, 1H), 6.43–6.49 (m, 2H), 5.08 (s, 2H), 3.54 (s, 3H), 2.73 (s, 2H); ^13^C NMR (DMSO-d_6_, 100 MHz): *δ* = 173.05, 172.49, 137.10, 136.98, 80.39, 80.06, 51.88, 47.04, 46.33.

### 4.2. Synthesis of CPT (5-ene-2-methyloxycarbonyl) norcantharidin acid ester **4**


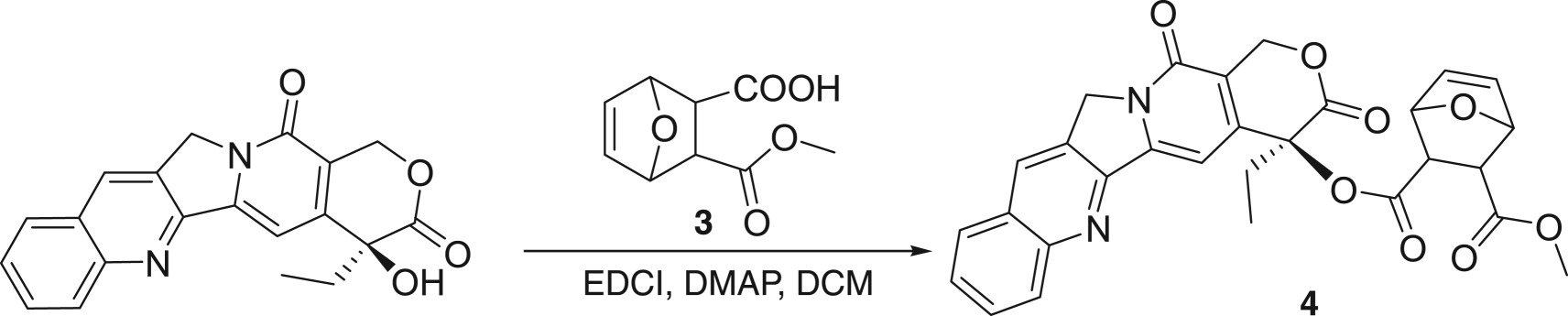


1.2 g (3.48 mmol) of camptothecin was dissolved in 20 ml of chloroform, followed by the addition of 5-ene-norcantharidin mono-methyl ester (**3**, 1.38 g, 7 mmol), EDCI (3.2 g, 16.8 mmol) and DMAP (240 mg, 2.44 mmol) in sequence. After 10 h, chloroform (30 ml) was added to dilute the solution, which was washed with water, saturated Na_2_CO_3_ solution and brine. The organic layer was dried over MgSO_4_. After the removal of the solvent, the residue was purified by chromatography through a silica gel column to afford the title compound (**4**, 1.62 g, 89%) as a yellow solid. Mp: 190–191°C, *R*_f_ = 0.40 (DCM/MeOH = 97/3). IR (KBr): *ν* (cm^−1^) = 3467, 3420, 3244, 2930, 1731, 1626, 1400, 1303, 1147, 612. ^1^H NMR (CDCl_3_, 400 MHz): *δ* = 8.40 (s, 1H), 8.20 (d, *J* = 8 Hz, 1H), 7.94 (d, *J* = 8 Hz, 1H), 7.84 (t, *J *= 8 Hz, 1H), 7.68 (t, *J *= 8 Hz, 1H), 7.19 (s, 1H), 7.0 (d, *J* = 16 Hz, 1H), 6.93 (d, *J *= 16 Hz, 1H), 5.71 (d, *J *= 24 Hz, 1H), 5.42 (d, *J *= 24 Hz, 1H), 5.30 (s, 2H), 3.83 (s, 3H), 2.95 (s, 1H), 2.88 (s, 1H), 2.17–2.38 (m, 2H), 1.69–1.78 (m, 2H), 1.01 (t, *J *= 8 Hz, 3H). ^13^C NMR (CDCl_3_, 100 MHz): *δ* = 167.38, 165.42, 164.08, 157.74, 152.63, 149.26, 146.89, 145.68, 135.77, 132.51, 131.69, 131.20, 130.02, 128.84, 128.66, 128.62, 128.57, 120.71, 96.25, 67.60, 53.00,50.44, 37.11, 32.27, 29.89, 27.56, 23.19, 14.67, 8.06. HRMS: Cal: 528.1533, Found: 529.1603 (M + 1).

### 4.3. Synthesis of side chain 5-ene norcantharidin monobenzyl ester **5**


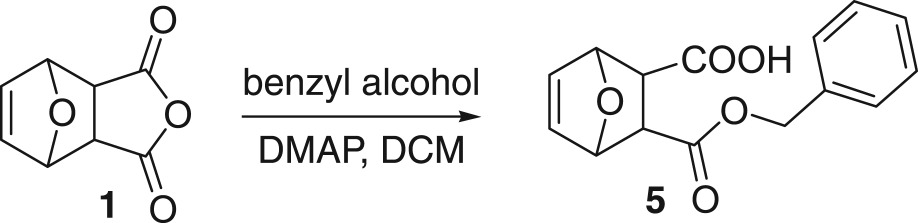


To a suspension of **1** (5 g, 30 mmol) in 40 ml of DCM was added benzyl alcohol (4.7 ml, 45 mmol) and DMAP (366 mg, 3 mmol). The mixture was stirred at room temperature for 3 days. The resulting precipitate was collected by filtration and dried under vacuum to afford the title product (**5**, 4.08 g, 50%) as a white solid. Mp: 129–130°C; ^1^H NMR (DMSO-d_6_, 400 MHz): *δ* = 12.43 (s, 1H), 7.30–7.35 (m, 5H), 6.43 (d, *J *= 4 Hz, 2H), 4.92–5.10 (m, 4H). ^13^C NMR (DMSO-d_6_, 100 MHz): *δ* = 173.07, 171.93, 137.15, 136.97, 136.41, 128.79, 128.27, 80.49, 80.16, 66.31, 47.14, 46.41.

### 4.4. Synthesis of CPT norcantharidin acid ester (**I**)


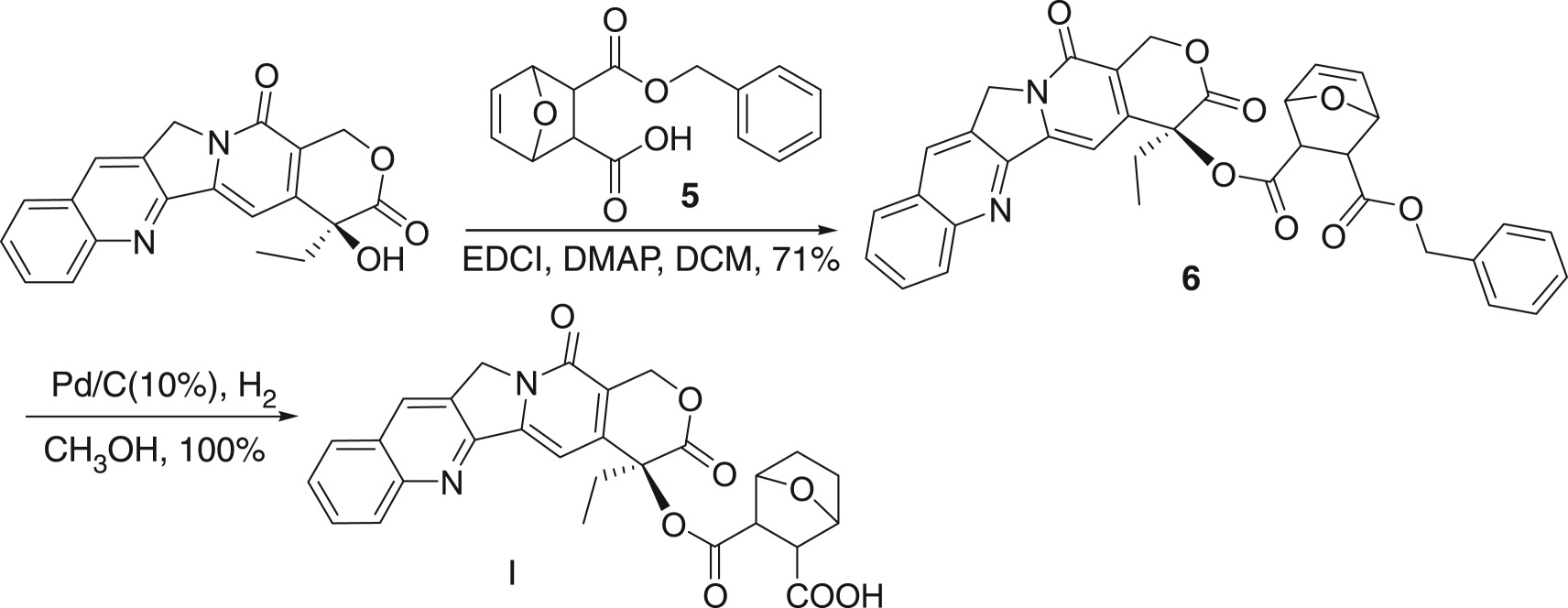


#### 4.4.1. Synthesis of CPT (5-ene-2-benzyloxycarbonyl) norcantharidin acid ester **6**

In a sealed tube was added 60 mg (0.17 mmol) of camptothecin and 10 ml of dichloromethane, followed by the addition of 5-ene-norcantharidin mono-benzyl ester (**5,** 93.2 mg, 0.34 mmol, 2 equ.), EDCI (161.3 mg, 0.84 mmol) and DMAP (13.4 mg, 0.11 mmol) in sequence. The mixture was heated to 50°C for 24 h. Dichloromethane (30 ml) was added to dilute the solution, which was washed with water, saturated Na_2_CO_3_ solution and brine. The organic layer was dried over MgSO_4_. After the removal of the solvent, the residue was purified by chromatography through a silica gel column to afford the title compound (**6**, 73.5 mg, 71.6%) as a yellow solid. Mp: 218–219°C, *R*_f_ = 0.44 (DCM/MeOH = 95/5). ^1^H NMR (CDCl_3_, 400 MHz): *δ* = 8.39 (s, 1H), 8.20 (d, *J *= 8 Hz, 1H), 7.94 (d, *J *= 8 Hz, 1H), 7.84 (d, *J *= 8 Hz, 1H), 7.81 (s, 1H), 7.67 (t, *J *= 8 Hz, 1H), 7.38 (m, 5H), 7.18 (s, 1H), 6.99 (d, *J *= 4 Hz, 2H), 5.71 (d, *J *= 16 Hz, 1H), 5.42 (d, *J *= 16 Hz, 1H), 5.27 (d, *J *= 12 Hz, 1H), 2.18–2.34 (m, 2H), 1.07–1.12 (m, 2H), 1.01 (t, *J *= 8 Hz, 3H). ^13^C NMR (CDCl_3_, 100 MHz): *δ* = 165.84, 163.27, 162.53, 156.27, 151.16, 147.82, 145.41, 144.22, 134.38, 133.94, 131.22, 130.19, 129.61, 128.43, 127.66, 127.57, 127.39, 127.17, 127.16, 127.07, 119.26, 108.97, 94.74, 66.31, 66.12, 48.95, 36.07, 30.81, 28.68, 21.67, 13.40, 6.58.

#### 4.4.2. Synthesis of target compound **I**

The benzyl ester **6** (500 mg) was dissolved in methanol (10 ml) and hydrogenated at atmospheric pressure, upon the treatment of palladium on carbon (50 mg, 10%) at ambient temperature for 2 h. The catalyst was removed by filtration. The filtrate was evaporated completely under reduced pressure. The residue was purified by chromatography, eluting with CH_2_Cl_2_/methanol = to give the title product as a yellow solid (427 mg, 100%). Mp: 209–210°C; *R*_f_ = 0.29 (DCM/MeOH = 10/1); IR (KBr): *ν* (cm^−1^) = 3415, 3139, 1746, 1623, 1400, 1224, 1160. ^1^H NMR (DMSO-d_6_, 400 MHz): *δ* = 8.67 (s, 1H), 8.17 (d, *J* = 8 Hz, 1H), 8.11 (d, *J* = 8 Hz, 1H), 7.87 (t, *J* = 8 Hz, 1H), 7.71 (t, *J* = 8 Hz, 1H), 7.13 (s, 1H), 5.49 (t, *J* = 16 Hz, 2H), 5.27 (t, *J* = 20 Hz, 2H), 2.69–2.85 (m, 2H), 2.45–2.51 (m, 4H), 2.16 (d, *J* = 4 Hz, 2H), 1.23 (s, 2H), 0.92 (t, *J* = 8 Hz, 3H). ^13^C NMR (DMSO-d_6_, 100 MHz): *δ* = 173.68, 171.81, 167.64, 156.95, 152.79, 148.29, 146.33, 145.71, 131.97, 130.83, 130.18, 129.41, 128.95, 128.37, 128.13, 119.33, 95.56, 76.28, 66.73, 50.62, 30.82, 29.13, 29.08, 7.99 (four carbons are missing in the carbon spectrum maybe because of the overlap). HRMS: Cal: 516.4988, Found: 471.1159 (M+1-CO_2_).

*Proliferation inhibition assay* HepG2, SW480, BGC803 and PANC-1 cells were cultured in RPMI 1640 or McCoy's 5A medium (Invitrogen), respectively, supplemented with 10% heat-inactivated FBS and 1% penicillin/streptomycin (Thermo Fisher Scientific). All cell lines were maintained at 37°C with 5% CO_2_. Cell viability was evaluated by the MTT assay. Cells were seeded in 96-well plates and incubated for 24 h. A range of concentrations of the test compounds were added and the plates were incubated for 72 h before the addition of 10 µl MTT (5 mg ml^−1^)/well. After 4 h of incubation, the medium was removed and 100 µl DMSO was added to each well. The absorbance was measured using a SpectraMax M5 microplate reader at 550 nm (table 1).

**Table 1.** *In vitro* antitumour activities (inhibition/%) of camptothecin analog **I**.

entry	HepG2	SW480	BGC803	PANC-1
solvent^a^	1.16	1.02	0.93	1.04
cantharidin	78.08	75.04	75.33	77.21
camptothecin	74.19	71.04	73.95	73.88
compound **I**	72.3	69.77	64.39	28.83

^a^test solvent DMSO.

A novel 20(*S*)-*O*-norcantharidin camptothecin derivative **I** was smoothly synthesized and it exhibited similar activities against HepG2 cell line. The SAR is being investigated and will be reported in the future.

## Supplementary Material

Supporting information

## Data accessibility

Data are available as part of the electronic supplementary material.
