# Supplementary figures and images for: A simple route to a novel acid-sensitive 20(S)-O-linked camptothecin norcantharidin acid ester derivative
Source: R Soc Open Sci. 2018 Feb 14;5(2):170842. doi: 10.1098/rsos.170842 (PMC5830714; doi:10.1098/rsos.170842)

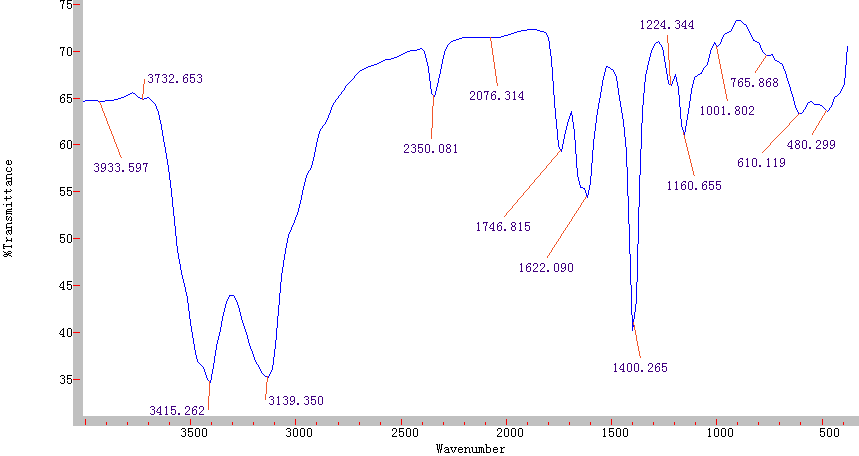


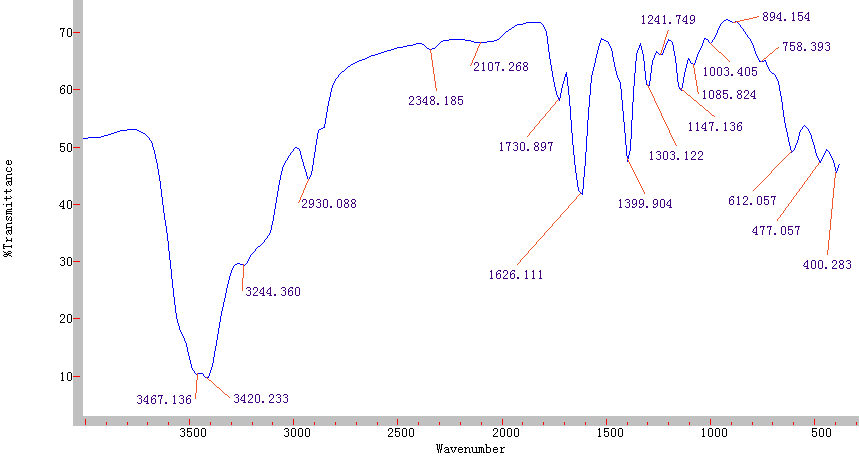

Supplement: Supporting information [file rsos170842supp1.doc]
